# Supplementary material for: OpenMM 7: Rapid development of high performance algorithms for molecular dynamics
Source: PLoS Comput Biol. 2017 Jul 26;13(7):e1005659. doi: 10.1371/journal.pcbi.1005659 (PMC5549999; doi:10.1371/journal.pcbi.1005659)
Supplement: S1 Source Code — (ZIP) [file pcbi.1005659.s002.zip › OpenMM-7.0.1-Source/docs/api-python/generated/simtk.openmm.openmm.AmoebaVdwForce.html]

AmoebaVdwForce — OpenMM 7.0.1.dev-5e86c4f documentation


# AmoebaVdwForce¶

*class* `simtk.openmm.openmm.``AmoebaVdwForce`(*\*args*)¶
:   This class implements a buffered 14-7 potential used to model van der Waals forces.

    To use it, create an AmoebaVdwForce object then call addParticle() once for each particle. After a particle has been added, you can modify its force field parameters by calling setParticleParameters(). This will have no effect on Contexts that already exist unless you call updateParametersInContext().

    A unique feature of this class is that the interaction site for a particle does not need to be exactly at the particle’s location. Instead, it can be placed a fraction of the distance from that particle to another one. This is typically done for hydrogens to place the interaction site slightly closer to the parent atom. The fraction is known as the “reduction factor”, since it reduces the distance from the parent atom to the interaction site.

    `__init__`(*self*) → AmoebaVdwForce¶
    :   \_\_init\_\_(self, other) -> AmoebaVdwForce

        Create an Amoeba VdwForce.

    Methods

    |  |  |
    | --- | --- |
    | `__init__`((self) -> AmoebaVdwForce) | \_\_init\_\_(self, other) -> AmoebaVdwForce |
    | `addParticle`((self, parentIndex, sigma, ...) | Add the force field parameters for a vdw particle. |
    | `getCutoff`((self) -> double) | Get the cutoff distance. |
    | `getEpsilonCombiningRule`(...) | Get epsilon combining rule |
    | `getForceGroup`((self) -> int) | Get the force group this Force belongs to. |
    | `getNonbondedMethod`(...) | Get the method used for handling long range nonbonded interactions. |
    | `getNumParticles`((self) -> int) | Get the number of particles |
    | `getParticleExclusions`(self, particleIndex) | Get exclusions for specified particle |
    | `getParticleParameters`(self, particleIndex) | Get the force field parameters for a vdw particle. |
    | `getSigmaCombiningRule`(...) | Get sigma combining rule |
    | `getUseDispersionCorrection`((self) -> bool) | Get whether to add a contribution to the energy that approximately represents the effect of VdW interactions beyond the cutoff distance. |
    | `setCutoff`(self, cutoff) | Set the cutoff distance. |
    | `setEpsilonCombiningRule`(self, ...) | Set epsilon combining rule |
    | `setForceGroup`(self, group) | Set the force group this Force belongs to. |
    | `setNonbondedMethod`(self, method) | Set the method used for handling long range nonbonded interactions. |
    | `setParticleExclusions`(self, particleIndex, ...) | Set exclusions for specified particle |
    | `setParticleParameters`(self, particleIndex, ...) | Set the force field parameters for a vdw particle. |
    | `setSigmaCombiningRule`(self, sigmaCombiningRule) | Set sigma combining rule |
    | `setUseDispersionCorrection`(self, useCorrection) | Set whether to add a contribution to the energy that approximately represents the effect of VdW interactions beyond the cutoff distance. |
    | `updateParametersInContext`(self, context) | Update the per-particle parameters in a Context to match those stored in this Force object. |
    | `usesPeriodicBoundaryConditions`((self) -> bool) | Returns whether or not this force makes use of periodic boundary conditions. |

    Attributes

    |  |  |
    | --- | --- |
    | `CutoffPeriodic` |  |
    | `NoCutoff` |  |

    `getNumParticles`(*self*) → int¶
    :   Get the number of particles

    `setParticleParameters`(*self*, *particleIndex*, *parentIndex*, *sigma*, *epsilon*, *reductionFactor*)¶
    :   Set the force field parameters for a vdw particle.

        |  |  |
        | --- | --- |
        | Parameters: | - **particleIndex** (*int*) – the particle index - **parentIndex** (*int*) – the index of the parent particle - **sigma** (*double*) – vdw sigma - **epsilon** (*double*) – vdw epsilon - **reductionFactor** (*double*) – the fraction of the distance along the line from the parent particle to this particle at which the interaction site should be placed |

    `getParticleParameters`(*self*, *particleIndex*)¶
    :   Get the force field parameters for a vdw particle.

        |  |  |
        | --- | --- |
        | Parameters: | **particleIndex** (*int*) – the particle index |
        | Returns: | - **parentIndex** (*int*) –   the index of the parent particle - **sigma** (*double*) –   vdw sigma - **epsilon** (*double*) –   vdw epsilon - **reductionFactor** (*double*) –   the fraction of the distance along the line from the parent particle to this particle at which the interaction site should be placed |

    `addParticle`(*self*, *parentIndex*, *sigma*, *epsilon*, *reductionFactor*) → int¶
    :   Add the force field parameters for a vdw particle.

        |  |  |
        | --- | --- |
        | Parameters: | - **parentIndex** (*int*) – the index of the parent particle - **sigma** (*double*) – vdw sigma - **epsilon** (*double*) – vdw epsilon - **reductionFactor** (*double*) – the fraction of the distance along the line from the parent particle to this particle at which the interaction site should be placed |
        | Returns: | index of added particle |
        | Return type: | int |

    `setSigmaCombiningRule`(*self*, *sigmaCombiningRule*)¶
    :   Set sigma combining rule

        |  |  |
        | --- | --- |
        | Parameters: | **sigmaCombiningRule** (*string*) – sigma combining rule: ‘ARITHMETIC’, ‘GEOMETRIC’. ‘CUBIC-MEAN’ |

    `getSigmaCombiningRule`(*self*) → std::string const &¶
    :   Get sigma combining rule

        |  |  |
        | --- | --- |
        | Returns: | sigmaCombiningRule sigma combining rule: ‘ARITHMETIC’, ‘GEOMETRIC’. ‘CUBIC-MEAN’ |
        | Return type: | string |

    `setEpsilonCombiningRule`(*self*, *epsilonCombiningRule*)¶
    :   Set epsilon combining rule

        |  |  |
        | --- | --- |
        | Parameters: | **epsilonCombiningRule** (*string*) – epsilon combining rule: ‘ARITHMETIC’, ‘GEOMETRIC’. ‘HARMONIC’, ‘HHG’ |

    `getEpsilonCombiningRule`(*self*) → std::string const &¶
    :   Get epsilon combining rule

        |  |  |
        | --- | --- |
        | Returns: | epsilonCombiningRule epsilon combining rule: ‘ARITHMETIC’, ‘GEOMETRIC’. ‘HARMONIC’, ‘HHG’ |
        | Return type: | string |

    `getUseDispersionCorrection`(*self*) → bool¶
    :   Get whether to add a contribution to the energy that approximately represents the effect of VdW interactions beyond the cutoff distance. The energy depends on the volume of the periodic box, and is only applicable when periodic boundary conditions are used. When running simulations at constant pressure, adding this contribution can improve the quality of results.

    `setUseDispersionCorrection`(*self*, *useCorrection*)¶
    :   Set whether to add a contribution to the energy that approximately represents the effect of VdW interactions beyond the cutoff distance. The energy depends on the volume of the periodic box, and is only applicable when periodic boundary conditions are used. When running simulations at constant pressure, adding this contribution can improve the quality of results.

    `setParticleExclusions`(*self*, *particleIndex*, *exclusions*)¶
    :   Set exclusions for specified particle

        |  |  |
        | --- | --- |
        | Parameters: | - **particleIndex** (*int*) – particle index - **exclusions** (*vector< int >*) – vector of exclusions |

    `getParticleExclusions`(*self*, *particleIndex*)¶
    :   Get exclusions for specified particle

        |  |  |
        | --- | --- |
        | Parameters: | **particleIndex** (*int*) – particle index |
        | Returns: | **exclusions** – vector of exclusions |
        | Return type: | vector< int > |

    `setCutoff`(*self*, *cutoff*)¶
    :   Set the cutoff distance.

    `getCutoff`(*self*) → double¶
    :   Get the cutoff distance.

    `getNonbondedMethod`(*self*) → OpenMM::AmoebaVdwForce::NonbondedMethod¶
    :   Get the method used for handling long range nonbonded interactions.

    `setNonbondedMethod`(*self*, *method*)¶
    :   Set the method used for handling long range nonbonded interactions.

    `updateParametersInContext`(*self*, *context*)¶
    :   Update the per-particle parameters in a Context to match those stored in this Force object. This method provides an efficient method to update certain parameters in an existing Context without needing to reinitialize it. Simply call setParticleParameters() to modify this object’s parameters, then call updateParametersInContext() to copy them over to the Context.

        The only information this method updates is the values of per-particle parameters. All other aspects of the Force (the nonbonded method, the cutoff distance, etc.) are unaffected and can only be changed by reinitializing the Context.

    `usesPeriodicBoundaryConditions`(*self*) → bool¶
    :   Returns whether or not this force makes use of periodic boundary conditions.

        |  |  |
        | --- | --- |
        | Returns: | true if nonbondedMethod uses PBC and false otherwise |
        | Return type: | bool |

    `__delattr__`¶
    :   x.\_\_delattr\_\_(‘name’) <==> del x.name

    `__format__`()¶
    :   default object formatter

    `__getattribute__`¶
    :   x.\_\_getattribute\_\_(‘name’) <==> x.name

    `__hash__`¶

    `__reduce__`()¶
    :   helper for pickle

    `__reduce_ex__`()¶
    :   helper for pickle

    `__sizeof__`() → int¶
    :   size of object in memory, in bytes

    `__str__`¶

    `getForceGroup`(*self*) → int¶
    :   Get the force group this Force belongs to.

    `setForceGroup`(*self*, *group*)¶
    :   Set the force group this Force belongs to.

        |  |  |
        | --- | --- |
        | Parameters: | **group** (*int*) – the group index. Legal values are between 0 and 31 (inclusive). |

High performance molecular simulation on GPUs


### Navigation

- Application Layer
- Library Layer
  - Core Objects
  - Forces
  - Integrators
  - Extras

©2015, Stanford University and the Authors.
|
Powered by Sphinx 1.3.1
& Alabaster 0.7.7
|
Page source
